# Southern Ocean Echinoids database – An updated version of Antarctic, Sub-Antarctic and cold temperate echinoid database

**DOI:** 10.3897/zookeys.697.14746

**Published:** 2017-09-14

**Authors:** Salomé Fabri-Ruiz, Thomas Saucède, Bruno Danis, Bruno David

**Affiliations:** 1 UMR 6282 Biogéosciences, Univ. Bourgogne Franche-Comté, CNRS, 6 bd Gabriel F-21000 Dijon, France; 2 Marine Biology Lab, CP160/15 Université Libre de Bruxelles, 50 avenue FD Roosevelt B-1050 Brussels, Belgium; 3 Muséum national d’Histoire naturelle, 57 rue Cuvier, 75005 Paris, France

**Keywords:** Echinoidea, oceanographic features, Southern Ocean, Antarctic, Sub-Antarctic

## Abstract

This database includes over 7,100 georeferenced occurrence records of sea urchins (*Echinodermata*: *Echinoidea*) obtained from samples collected in the Southern Ocean (+180°W/+180°E; -35°/-78°S) during oceanographic cruises led over 150 years, from 1872 to 2015. Echinoids are common organisms of Southern Ocean benthic communities. A total of 201 species is recorded, which display contrasting depth ranges and distribution patterns across austral provinces and bioregions. Echinoid species show various ecological traits including different nutrition and reproductive strategies. Information on taxonomy, sampling sites, and sampling sources are also made available.

Environmental descriptors that are relevant to echinoid ecology are also made available for the study area (-180°W/+180°E; -45°/-78°S) and for the following decades: 1955–1964, 1965–1974, 1975–1984, 1985–1994 and 1995–2012. They were compiled from different sources and transformed to the same grid cell resolution of 0.1° per pixel. We also provide future projections for environmental descriptors established based on the Bio-Oracle database (Tyberghein et al. 2012).

## Project description

**Project title**: Species distribution modelling of Echinoids in the Southern Ocean

**Personnel**: Salomé Fabri-Ruiz, Thomas Saucède, Bruno Danis, Bruno David

### Funding

SF-R’s work is supported by a PhD grant from French Ministry of Higher Education and Research. The project is a contribution to Biogeosciences laboratory (CNRS UMR6292) and contribution #15 to the vERSO project (www.versoproject.be) funded by the Belgian Science Policy Office (BELSPO, contract n°BR/132/A1/vERSO).

### Study area descriptions/ descriptors

The study area extends from the Antarctic continent in the south to 35° S latitude to the north; it comprises the sub-Polar, Antarctic, Polar Frontal, and sub-Antarctic zones. The Southern Ocean is characterized by unique oceanographic features mainly including an unusually deep continental shelf ranging from 450 m to 1000 m depth (Clarke and Johnston 2003), and the Antarctic Circumpolar Current (ACC), the strongest and largest current of the planet that flows clockwise from west to east around Antarctica (Barker and Thomas 2004) and conditions marine species dispersal (Griffiths et al. 2009). Four major marine fronts are distributed from north to south : Subtropical Front (STF), Sub-Antarctic Front (SAF), Polar Front (PF), Antarctic Divergence (AD), and separate water masses of different physical and biotic properties (Sokolov and Rintoul 2002, Roquet et al. 2009).

One of these major fronts is the Polar Front that acts as a biogeographic barrier to the dispersal of many invertebrates between sub-Antarctic and Antarctic waters (Koubbi 1993, Clarke et al. 2005).

### Design description

Nowadays, ecological niche modelling is commonly used in macroecological and biogeographic studies to enhance mapping and understanding of species distribution patterns. Models also constitute useful tools for marine area management purposes (Sánchez-Carnero et al. 2016), predicting invasive species distribution (Václavík and Meentemeyer 2012), identifying biodiversity hot spots and highlighting potential impacts of climate change on species distribution (Elith and Leathwick 2009). Extensive and consistent databases are essential to biogeographic studies to explore species distribution patterns in the Southern Ocean (De Broyer and Koubbi 2014). Reliability and robustness of distribution models are mainly conditioned by the quality and accuracy of occurrence data (Graham et al. 2007, Lobo 2008, Osborne and Leitão 2009). With this in mind, the creation of SCAR-Marbin in 2005 (Griffiths et al. 2011) and RAMS in 2010 (De Broyer and Danis 2011) allowed the first Antarctic marine biodiversity data compilation.

Objectives of our project are to produce robust and reliable species distribution models at the scale of the Southern Ocean, an area where distribution data are very heterogeneous and sampling gaps frequent.

This requires consistent and comprehensive datasets. For this purpose, an extensive echinoid occurrence dataset was compiled, updated, and checked for accuracy. This dataset is presented here.

Taxonomic information was updated according to the most recent literature. For example, *Sterechinus bernasconiae* Larrain, 1975 is now considered a junior synonym of *Gracilechinus multidentatus* (Clark, 1925) (Saucède et al. 2015). We checked for taxonomic accuracy using the World Echinoidea database (Kroh and Mooi 2017) and experts knowledge. However, mentions of former species identifications are kept in the dataset and clearly distinguished from updated taxonomy.

**Figure 1.**
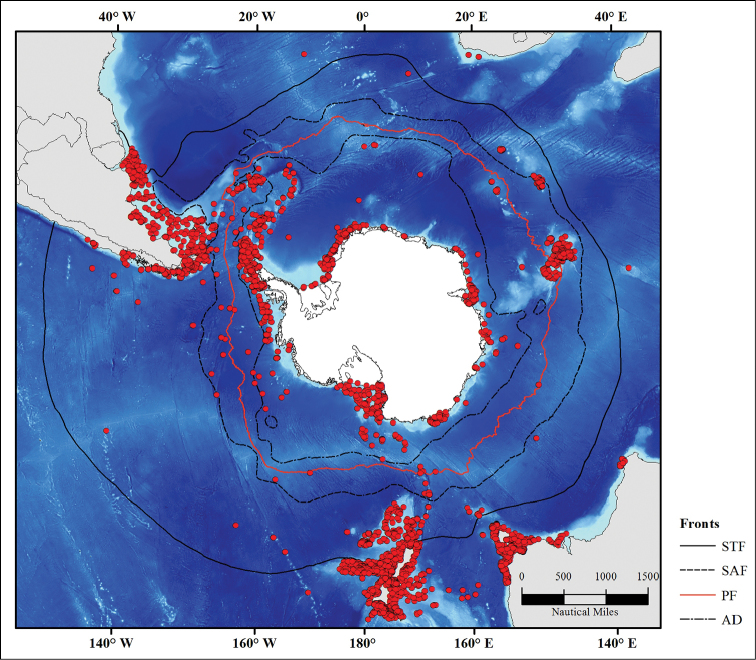
Echinoid occurrence records in the Southern Ocean with major marine fronts

The dataset includes historical data sampled in the Southern Ocean over a century and a half from the Challenger expedition to the most recent oceanographic campaigns led on the Kerguelen Plateau, in Adelie Land and around the Antarctic Peninsula (Figure 1). All compiled georeferenced locations were scanned and checked for accuracy.

*DatasetName* links the origins of occurrence records which are from academic collections (British Antarctic Survey Collection, Burgundy University collection, …), published articles, former databases (David et al. 2005, Pierrat et al. 2012) or cruise reports.

In order to quantify sampling effort, a 3° by 3° cell grid was shaped (Clarke et al. 2007) and each record of the database was assigned to a grid cell. Following Griffiths et al. (2011) sample and species numbers were both counted for each grid cell using ArcGIS v10.2 (ESRI 2011) and Microsoft Access (2013).

We also provide oceanographic features as environmental maps for physical and abiotic parameters that are relevant to echinoid ecology. Environmental data come from the World Ocean Circulation Experiment 2013 database and depth data come from ETOPO1 (Amante and Eakins 2009). Cell resolution was set up at 0.1 degree with the R 3.3.0 software. These data needed to be corrected for precise depth accuracy, which was performed using ArcGIS and following the protocol proposed by Guillaumot et al. (2016). A seafloor temperature layer was generated based on available temperatures for multiple depth layers of the water column. However, due to missing data, some values were interpolated using the nearest neighbour method with Arctoolbox (ESRI 2011).

### Sampling effort and data description

The database includes more than 7,100 georeferenced records (Figure 1). It is an updated version of the former database “Antarctic, Sub-Antarctic and cold temperate echinoid database” (Pierrat et al. 2012) that contains 1,000 additionnal records compared to Pierrat et al. 2012. This new version includes new records from the most recent oceanographic campaigns led in the Southern Ocean (e.g. POKER II, PROTEKER, ANT-XXIX/3) and recent reviews of academic collections (e.g. Smithsonian Institution Museums). In addition, taxonomy and georeferenced positions were updated and checked for accuracy. records.

Sampling effort has long been heterogeneous in the Southern Ocean. It has been the highest along the Antarctic Peninsula and off New Zealand (>200 samples), two areas characterized by a high species number (25–30) (Figure 2a, 2b). In contrast, the number of species remains low (2–5 species) in the region of the Kerguelen Plateau while it has been intensively sampled as well (POKER 2 and PROTEKER cruises).

**Figure 2a.**
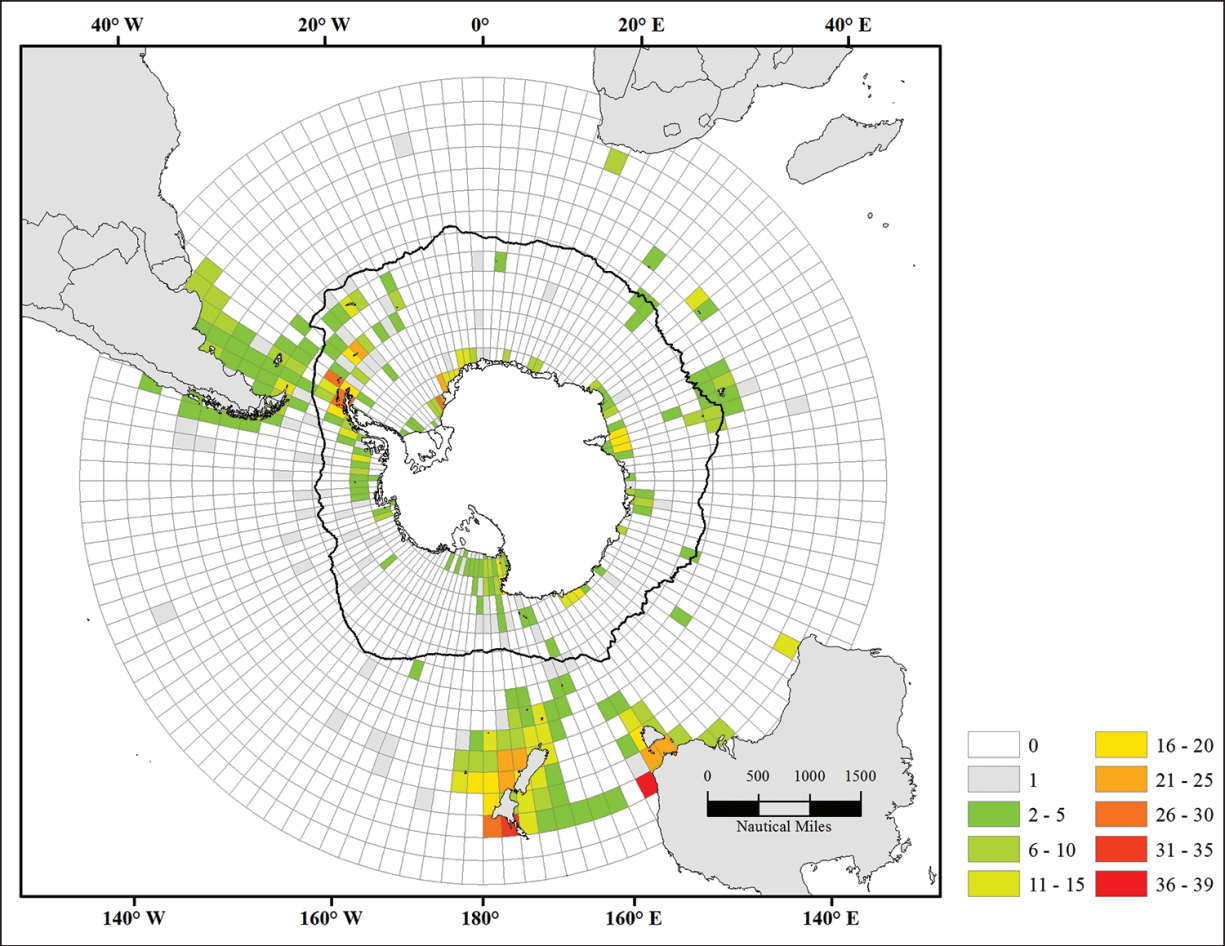
Number of species recorded per grid cell

**Figure 2b.**
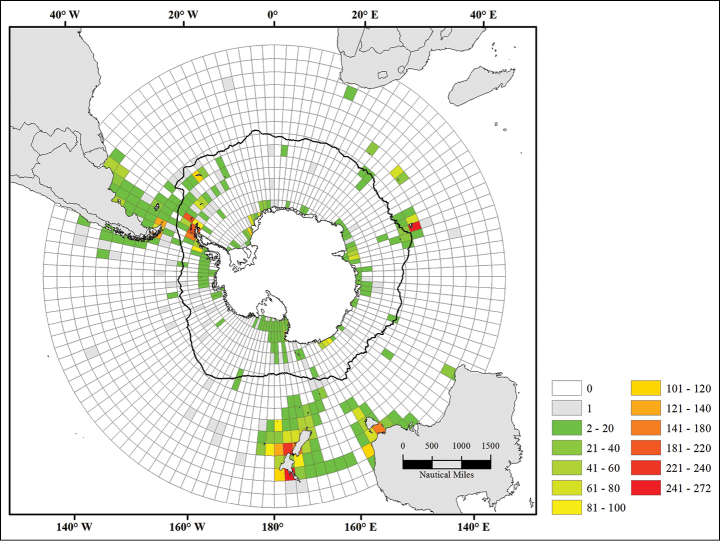
Number of samples recorded per grid cell

**Figure 3.**
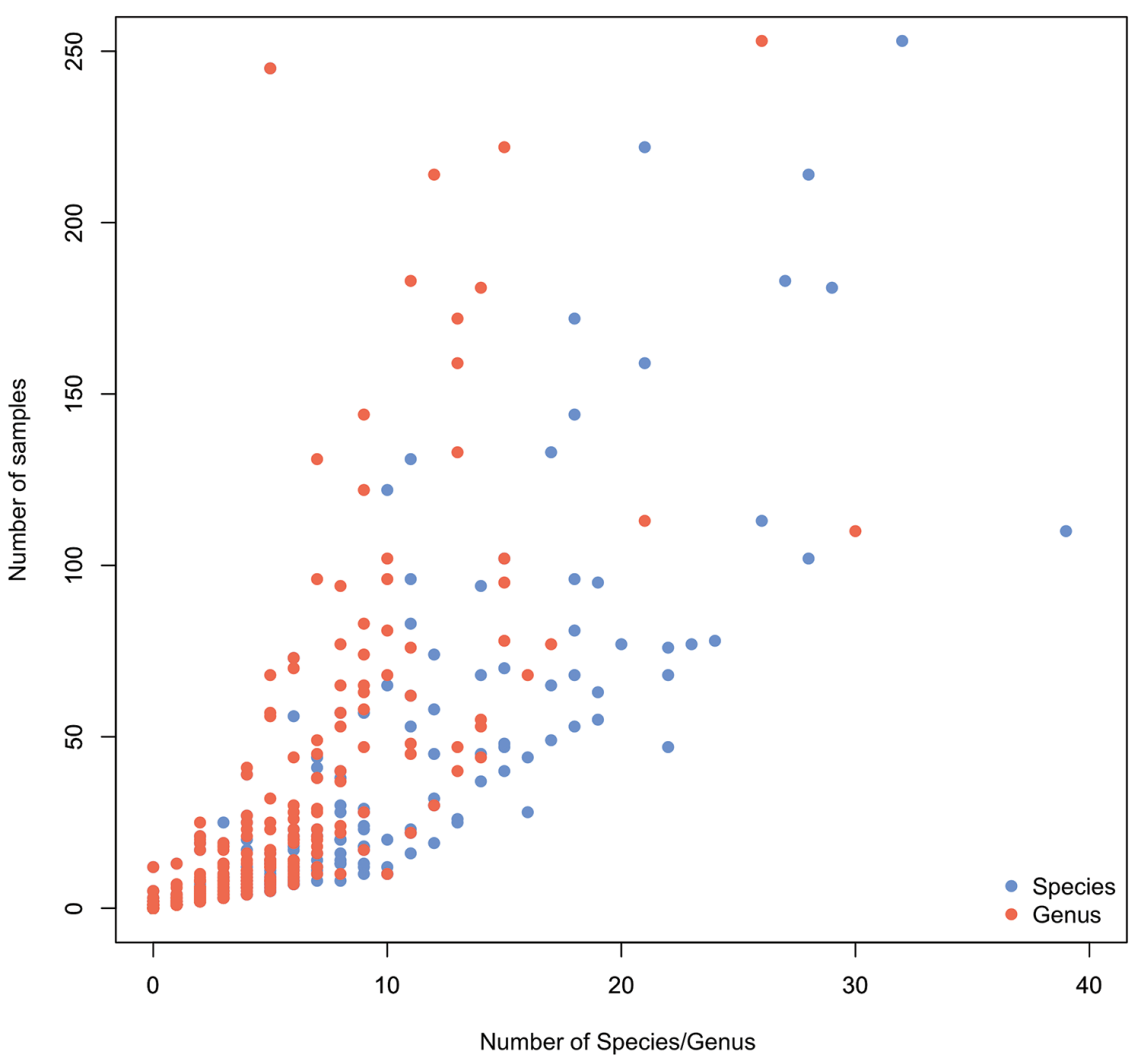
Number of recorded samples against species (blue dots) and genus (red dots) richness per grid cell in the Southern Ocean.

Our knowledge of genus and species distributions is strongly biased by the quality of sampling effort. Figure 3 highlights the link between the number of samples available and the recorded number of species and genera per grid cell.

**Figure 4.**
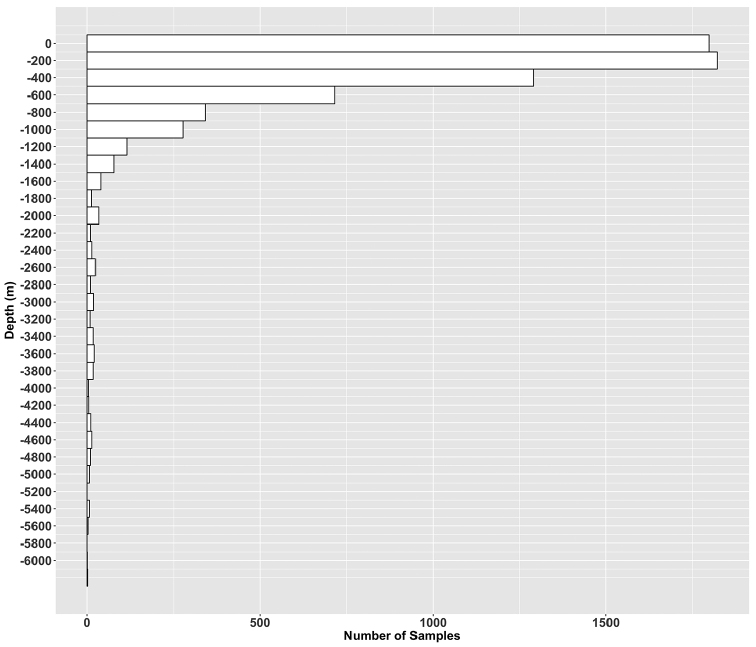
Number of occurrences according to depth (m)

Several areas have been little sampled including the waters close to the sea ice margin and deep oceanic basins, most records being concentrated in the first 400 meters (Figure 4) and in the vicinity of scientific stations like in the north of the Kerguelen Plateau, in Adelie Land or along the Antarctic Peninsula. Conversely, the South Kerguelen Plateau and the west of the Ross Sea have been little explored. These under-sampled parts of the Southern Ocean constitute challenging areas for future scientific cruises. However, new sampling technics and standardizations over the last few years improved our knowledge of the Southern Ocean biodiversity (Kaiser et al. 2013). Common tools have been developed like ecological niche modelling in order to interpolate occurrence records to under-sampled areas and allow improving our knowledge of species potential distribution areas.

### Latitudinal gradient

**Figure 5.**
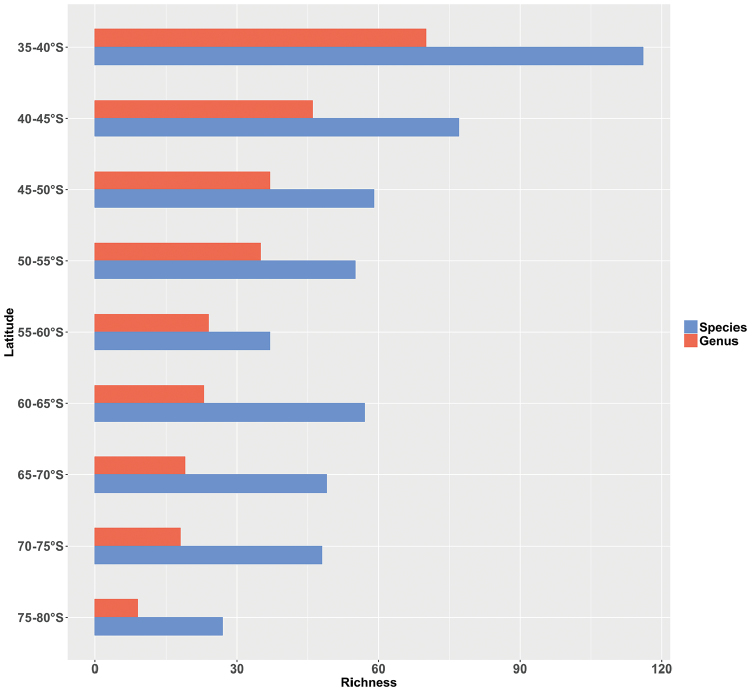
Species (blue) and genus (red) richness against latitude

Main biogeographic features of Southern Ocean echinoids is a constant decrease of genus richness southward whereas species richness decreases from 35°S to 60°S, increases from 60°s to 65°S, then decreases again southward until 70°S (Figure 5). Such a pattern has already been published for Southern Ocean echinoids (Saucède et al. 2014) and herein supported by new data addition. The high number of species recorded between 60°S and 65°S could be due to the high sampling effort devoted to the region of the Antarctic Peninsula (Figure 2a–b) while conversely, sampling effort decreases southward until 70°S.

### Environmental data

Environmental data were compiled from the following sources: Smith and Sandwell 1997, Boyer et al. 2013, Douglass et al. 2014. Environmental data are provided in raster format (Fabri-Ruiz et al, 2017). Mean surface temperature, mean seafloor temperature, mean surface salinity, and their respective amplitudes (winter minus summer averages) were calculated for the following decades: [1955 to 1964], [1965 to 1974], [1975 to 1984], [1985 to 1994] and [1995 to 2012]. Future projections are provided for mean surface temperature and salinity and for different IPCC scenarios (A2, A1B, B1) (IPCC, 5^th^) they were downloaded from the Bio-Oracle database (Tyberghein et al. 2012).

## Taxonomic coverage

### General taxonomic coverage

The database includes occurrence records of all echinoid species reported in the Southern Ocean from the Antarctic continent to 35°S latitude. Echinoids are common organisms of Southern Ocean benthic communities. They have contrasting depth ranges and distribution patterns across austral provinces and bioregions, ranging from coastal areas to the abyssal zone. Echinoid species show various ecological traits including different nutrition and reproductive strategies. In total, 201 species belonging to 31 families were recorded. Many of them are endemic to the Southern Ocean.

### Taxonomic ranks

**Kingdom**: Animalia

**Phylum**: Echinodermata

**Class**: Echinoidea

**Order**: Arbacioida, Camarodonta, Cassiduloida, Cidaroida, Clypeasteroida, Diadematoida, Echinoida, Echinothurioida, Holasteroida, Neognathostomata, Pedinoida, Salenioida, Spatangoida, Temnopleuroida.

**Family**: Arbaciidae, Arachnoididae, Arbaciidae, Aspidodiadematidae, Asterostomatidae, Brissidae, Clypeasteridae, Ctenocidarinae, Cyclasterinae, Diadematidae, Echinidae, Echinolampadidae, Echinometridae, Echinothuriidae, Fibulariidae, Hemiasteridae, Kamptosomatidae, Laganidae, Loveniidae, Mellitidae, Palaeotropidae, Pedinidae, Phormosomatidae, Plexechinidae, Pourtalesiidae, Saleniidae, Schizasteridae, Spatangidae, Temnopleuridae, Toxopneustidae, Urechinidae.

**Genus**: *Abatus*, *Aceste*, *Amblypneustes*, *Ammotrophus*, *Amphipneustes*, *Anametalia*, *Antrechinus*, *Apatopygus*, *Aporocidaris*, *Araeosoma*, *Arbacia*, *Aspidodiadema*, *Austrocidaris*, *Brachysternaster*, *Brisaster*, *Brissopsis*, *Brissus*, *Caenocentrotus*, *Caenopedina*, *Calveriosoma*, *Centrostephanus*, *Ceratophysa*, *Clypeaster*, *Coelopleurus*, *Ctenocidaris*, *Cyclaster*, *Cystechinus*, *Cystocrepis*, *Delopatagus*, *Dermechinus*, *Diadema*, *Echinocardium*, *Echinocrepis*, *Echinocyamus*, *Echinolampas*, *Echinosigra*, *Echinus*, *Encope*, *Eupatagus*, *Evechinus*, *Fellaster*, *Fibularia*, *Genicopatagus*, *Goniocidaris*, *Gracilechinus*, *Gymnopatagus*, *Helgocystis*, *Heliocidaris*, *Hemiaster*, *Heterobrissus*, *Histocidaris*, *Holopneustes*, *Hygrosoma*, *Kamptosoma*, *Linopneustes*, *Loxechinus*, *Mellita*, *Microcyphus*, *Moira*, *Notocidaris*, *Ogmocidaris*, *Orechinus*, *Pachycentrotus*, *Paleotrema*, *Paramaretia*, *Peronella*, *Phormosoma*, *Phyllacanthus*, *Pilematechinus*, *Plexechinus*, *Polyechinus*, *Poriocidaris*, *Pourtalesia*, *Prionocidaris*, *Protenaster*, *Pseudechinus*, *Pseudoboletia*, *Rhopalocidaris*, *Rhynchocidaris*, *Salenia*, *Salenocidaris*, *Salmaciella*, *Solenocystis*, *Spatagocystis*, *Spatangus*, *Sperosoma*, *Sterechinus* , *Stereocidaris*, *Stylocidaris*, *Temnopleurus*, *Tetrapygus*, *Toxopneustes*, *Tripneustes*, *Tripylaster*, *Tripylus*, *Tromikosoma*, *Urechinus*.

### Spatial coverage

**General spatial coverage**: Southern Ocean

**Coordinates**: 79°0'0"S and 35°0'0"S Latitude; 180°0'0"W and 180°0'0"E Longitude

### Temporal coverage

Temporal coverage: 1872–2015

## Datasets

### Dataset occurrence description

Occurrence of echinoids in the Southern Ocean from 1872 to 2015.

**Object name**: Echinoids_occurrences_Southern_Ocean

**Character encoding**: x-MacRoman

**Format name**: Darwin Core Archive Format

**Distribution**: http://ipt.biodiversity.aq/resource?r=echinoids_occurrences_southern_ocean

**Publication of data**: 2017-06-22

**Language**: English

**Metadata language**: English

**Date of metadata creation**: 2017-06-22

**Hierarchy level**: Dataset

### Environmental parameters description

Environmental descriptors for the Southern Ocean were compiled from various sources but most of them come from the World Ocean Atlas (Boyer et al. 2013) for current parameters. Available data are mean surface temperature, mean seafloor temperature, mean surface salinity and their respective amplitudes (winter minus summer averages) were calculated for the following decades: [1955 to 1964], [1965 to 1974], [1975 to 1984], [1985 to 1994] and [1995 to 2012]. Future projections are provided for mean surface temperature and salinity and for different IPCC scenarios (A2, A1B, B1) (IPCC 5^th^); they were downloaded from the Bio-Oracle database (Tyberghein et al. 2012).

**Object name**: Environmental_data_Southern_Ocean

**Format name**: Raster

**Distribution**: data.aad.gov.au/metadata/records/Environmental_data_Southern_Ocean doi: 10.4225/15/5949ba54ca33c

**Publication date of data**: 2017-05-18

**Language**: English

**Metadata language**: English

**Date of metadata creation**: 2017-05-18

**Hierarchy level**: Dataset

## References, referred to in the datasets

Agassiz A (1863) List of the echinoderms sent to different institutions in exchange for other specimens, with annotations. Bulletin of the Museum of Comparative Zoölogy at Harvard College 1: 17–28.

Agassiz A (1864) Synopsis of the echinoids collected by Dr. W. Stimpson on the North Pacific Exploring Expedition under the command of Captains Ringgold and Rodgers. Proceedings of the Academy of Natural Sciences of Philadelphia 15: 352–361.

Agassiz A (1869) Preliminary report on the echini and star-fishes dredged in deep water between Cuba and the Florida Reef, by L. F. de Pourtalès, Assist. U.S. Coast Survey. Bulletin of the Museum of Comparative Zoölogy at Harvard College 1: 253–308.

Agassiz A (1872a) Preliminary notice of a few species of Echini. Bulletin of the Museum of Comparative Zoölogy at Harvard College 3: 55–58.

Agassiz A (1872b) 7 Revision of the Echini. Illustrated Catalogue of the Museum of Comparative Zoölogy at Harvard College, 378 pp.

Agassiz A (1878) Report on the results of dredging, under the supervision of Alexander Agassiz, in the Gulf of Mexico, by the U.S. Coast Survey Steamer “Blake”, Lieutenant-Commander C. D. Sigsbee, U.S.N., Commanding. Report on the Echini. Bulletin of the Museum of Comparative Zoölogy at Harvard College 5: 181–195.

Agassiz A (1879) Preliminary Report on the Echini of the Exploring Expedition of H. M. S. “Challenger,” Sir C. Wyville Thomson Chief of Civilian Staff. Proceedings of the American Academy of Arts and Sciences 6: 190–212.

Agassiz A (1881) Report on the Echinoidea dredged by HMS “Challenger” 1873-76. HMS Challenger 3: 1–321.

Agassiz A (1898) Reports on the dredging operations off the west coast of Central America to the Galapagos, to the west coast of Mexico and in the Gulf of California, in charge of Alexander Agassiz, carried on by the U.S. Fish Commission steamer “Albatross” during 1891, Lieut. Commander Z.L. Tanner, U.S.N., Commanding. XXIII. Preliminary report on the Echini. Bulletin of the Museum of Comparative Zoology 32: 71–86.

Agassiz A, Clark HLK (1907a) Preliminary report on the echini collected, in 1902, among the Hawaiian Islands, by the U.S. Fish Comussion steamer “Albatross”, in charge of Commander Chauncey Thomas, U.S.N., commanding. Bulletin of the Museum of Comparative Zoölogy at Harvard College 50: 231–259.

Agassiz A, Clark HLK (1907b) Preliminary report on the Echini collected in 1906, from May to December, among the Aleutian Islands, in Bering Sea, and along the coasts of Commission steamer “Albatross”, in charge of Lieut.-Commander. Bulletin of the Museum of Comparative Zoölogy at Harvard College 51: 107–139.

Agassiz L (1835) Podrome d’une monographie des radiaires ou échinodermes. Mémoires de la Société des Sciences Naturelles de Neuchâtel 1: 168–199.

Agassiz L (1838) Monographies d’Échinodermes vivants et fossiles. Première monographie: Des Salénies. Petitpierre, Neuchâtel, 32 pp. https://doi.org/10.5962/bhl.title.1833

Agassiz L (1840) Catalogus systematicus Ectyporum Echinodermatum fossilium Musei Neocomiensis, secundum ordinem zoologicum dispositus; adjectis synonymis recentioribus, nec non stratis et locis in quibus reperiuntur. Sequuntur characteres diagnostici generum novorum vel minus cognitorum. Petitpierre, Neuchâtel, 20 pp.

Agassiz L (1841) Monographies d’Echinodermes vivans et fossiles. Echinites. Famille des Clypéasteroides. Seconde Monographie. Des Scutelles, 149 pp. https://doi.org/10.5962/bhl.title.126954

Agassiz L (1846) Catalogue raisonné des familles, des genres, et des espèces de la classe des échinodermes. Annales des Sciences Naturelles, Troisième Série, Zoologie 6: 305–374.

Agassiz L (1847) Catalogue raisonné des espèces, des genres, et des familles d’échinides. Annales des Sciences Naturelles, Troisième Série, Zoologie 8: 355–380.

Alcock A (1893) Natural history notes from H.M. Indian Marine Survey steamer Investigator, Commander C.F. Oldham, R.N., commanding. Series 2, No 9. An account of the deep-sea collection made during the season of 1892–93. Journal of the Asiatic Society of Bengal 62: 169–184.

Anderson OF (2009) The giant purple pedinid—a new species of *Caenopedina* (Echinodermata: Echinoidea: Pedinidae) from New Zealand and Australia. Zootaxa 2007: 43–57.

Baker AN (1967) Two New Echinoids from Northern New Zealand, including a New Species of *Diadema*. Transactions of the Royal Society of New Zealand, Zoology 8: 239–245.

Baker AN (1968) A New Cidarid Echinoid from Northern New Zealand. Transactions of the Royal Society of New Zealand, Zoology 10: 199–203.

Baker AN (1969) Two new heart-urchins, including a new species of Cyclaster, from New Zealand waters (Echinoidea) Spatangoida. Records of the Dominion Museum 6: 265–273.

Baker AN, Rowe FWE (1990) Atelostomatid sea urchins from Australian and New Zealand waters (Echinoidea. Cassiduloida, Holasteroida, Spatangoida, Neolampadoida). Invertebrate Taxonomy 4: 281–316. https://doi.org/10.1071/IT9900281

Bell FJ (1880) On Palaeolampas, a new genus of the Echinoidea. Proceedings of the Zoological Society 4: 43–49.

Bell FJ (1884) Echinodermata: Report on the zoological collections made in the Indo-Pacific Ocean during the voyage of HMS «Alert» 1881–82. : 509–512.

Bell FJ (1904) On the Echinodermata found off the coast of South Africa. Part 1 *Echinoidea*. Marine Invest. S. Africa 3: 167–176.

Benham WB (1908) An Erroneous Echinodermal Identification. Journal of Natural History 1: 104–108. https://doi.org/10.1080/00222930808692363

Bernasconi I (1947) Una nueva especie de “Mellita” en la Republica Argentina. Physis 20: 117–118.

Blainville HMD (1825) Oursin, Echinus (Actinozoaires). In: Levrault FG (Ed.) Dictionnaire des Sciences Naturelles. Strasbourg & Paris, 59–98.

Blount C, Worthington D (2002) Identifying individuals of the sea urchin Centrostephanus rodgersii with high-quality roe in New South Wales, Australia. Fisheries Research 58: 341–348. https://doi.org/10.1016/S0165-7836(01)00399-X

Clark HLK (1909) Scientific results of the trawling expedition of H.M.C.S. “Thetis” off the coast of New South Wales, in February and March, 1898, Echinodermata. Australian Museum Memoir 4: 519–564. https://doi.org/10.3853/j.0067-1967.4.1909.1507

Clark HLK (1912) Hawaiian and other Pacific Echini. The Pedinidae, Phymosomatidae, Stomopneustidae, and Echinometridae. Memoirs of the Museum of Comparative Zoölogy at Harvard College 34: 205–383.

Clark HLK (1914) Hawaiian and other Pacific Echini. The Clypeasteridae, Arachnoididae, Laganidae, Fibulariidae, and Scutellidae. Memoirs of the Museum of Comparative Zoology at Harvard College 46: 1–80.

Clark HLK (1916) Report on the sea-lilies, starfishes, brittle-stars and sea-urchins obtained by the F.I.S. “Endeavour” on the coasts of Queensland, New South Wales, Tasmania, Victoria, South Australia, and Western Australia. Biological Results of the Fishing experiments carried on by the F.I.S. Endeavour 1909–1914 4: 1–123.

Clark HLK (1925) A catalogue of the recent sea-urchins (Echinoidea) in the collection of the British Museum (Natural History). Oxford Univ. Press, London, 250 pp.

Clark HLK (1928) The sea-lilies, sea-stars, brittle-stars and sea-urchins of the South Australian Museum. Records of the South Australian Museum 3: 361–482.

Clark HLK, Agassiz A (1907) Hawaiian and other Pacific Echini. The Cidaridae. Memoirs of the Museum of Comparative Zoology at Harvard College 34: 1–42.

Claus K (1880) 1 Grundzüge der Zoologie. N.G. Elwert’sche Verlagsbuchhandlung, Marburg, 821 pp.

Dartnall AJ (1972) A brooding echinoid from Tasmania. Proceedings of the Linnean Society of New South Wales, 30–34.

David B, Mooi R (1990) An echinoid that “gives birth”: morphology and systematics of a new Antarctic species, *Urechinus mortenseni* (Echinodermata, Holasteroida). Zoomorphology 110: 75–89. https://doi.org/10.1007/BF01632814

Deheyn DD, Gendreau P, Baldwin RJ, Latz MI (2005) Evidence for enhanced bioavailability of trace elements in the marine ecosystem of Deception Island, a volcano in Antarctica. Marine Environmental Research 60: 1–33. https://doi.org/10.1016/j.marenvres.2004.08.001

Delage Y, Hérouard E (1903) 3 Traité de zoologie concrète. Schleicher. Beinwald, Paris, 496 pp.

Desor E (1855) 1 Synopsis des échinides fossiles. Reinwald, Paris, 490 pp.

Döderlein LHP (1885) Seeigel von Japan und den Liu-Kiu Inseln. Archiv für Naturgeschichte 51: 73–112. https://doi.org/10.5962/bhl.part.1569

Döderlein LHP (1887) 1 Die japanischen Seeigel. I: Familien Cidaridae und Saleniidae. E. Schweizerbart’sche Verlagshandlung. Stuttgart, 59 pp.

Döderlein LHP (1905) Ueber Seeigel der deutschen Tiefsee-Expedition. Zoologischer Anzeiger 282: 621–624.

Döderlein LHP (1906) Die Echinoiden der Deutschen Tiefsee-Expedition. In: Chun C (Eds) Wissenschaftliche Ergebnisse der Deutschen Tiefsee-Expedition auf dem Dampfer “Valdivia” 1898–1899. Fischer, Jena. , 61–290. https://doi.org/10.5962/bhl.title.46999

Döderlein LHP (1914) Echinoidea. In: Michaelsen W, Hartmeyer R (Eds) Die Fauna Südwest-Australiens. Gustav Fischer, Jena, 443–492.

Dollfus RP (1936) Sur un *Pseudechinus* recolte par Charles Velain à I’Ile Saint Paul: Observations morphologiques et biogeographiques. Mémoires du Muséum National d’Histoire Naturelle 22: 160–178.

Duncan PM (1889) A Revision of the Genera and great Groups of the Echinoidea. Zoological Journal of the Linnean Society 23: 1–311. https://doi.org/10.1111/j.1096-3642.1889.tb01431.x

Durham JW, Melville RV (1957) A classification of echinoids. Journal of Paleontology 31: 242–272.

Fell HB (1958) Deep-sea echinoderms of New Zealand. Zoology Publications from Victoria University of Wellington 24: 1–40.

Fell HB (1963) The spatangid echinoids of New Zealand. Zoology Publications from Victoria University of Wellington 32: 1–8.

Forbes E (1841) A history of British Starfishes and other animals of the class Echinodermata. John Van Voorst, London, 267 pp. https://doi.org/10.5962/bhl.title.2129

Gray JE (1825) An attempt to divide the Echinida, or sea eggs, into natural families. Annals of Philosophy 10: 423–431.

Gray JE (1851) Description of two new genera and some new species of Scutellide and Echinolampide in the collection of the British Museum. Proceedings of the Zoological Society of London, 34–38. https://doi.org/10.1111/j.1096-3642.1851.tb01127.x

Gray JE (1855) An arrangement of the families of Echinida, with descriptions of some new genera and species. Proceedings of the Zoological Society. London, 35–39.

Gray JE (1855) Catalogue of the recent Echinida, or sea eggs: in the collection of the British museum. Part I. Echinida irregularia. Woodfall & Kinder, London, 69 pp.

Gregory JW (1900) The Echinoidea. In: Lankester ER (Ed.) A Treatise on Zoology. Part III. The Echinoderma, 282–332.

Grua P (1963) Etude de biotopes marins infralittoraux (Kerguelen 1962–63). TAAF, Paris, 69–73.

Hutton FW (1872) Catalogue of the echinodermata of New Zealand, with diagnoses of the species. Colonial Museum and Geological Department, 1–18.

Hutton FW (1878) Notes on some New Zealand Echinodermata, with descriptions of new species. Transactions and Proceedings of the New Zealand Institute 11: 305–308.

Kier PM (1962) Revision of the cassiduloid echinoids. Smithsonian Miscellaneous Collections 144: 1–262.

Kier PM (1984) The fossil spatangoid echinoids of Cuba. Smithsonian Contributions to Paleobiology 55: 1–134. https://doi.org/10.5479/si.00810266.55.1

Koehler (1914) 258 Échinides du Musée Indien à Calcutta. I. Spatangidés. Echinoderma of the Indian Museum Part 8, Echinoidea (1), 258 pp.

Koehler R (1897) *Sperosoma Grimaldii*, nouveau genre d’Echinothuride. Zoologischer Anzeiger 20: 302–307.

Koehler R (1900) Note préliminaire sur les Echinides et les Ophiures de l’Expédition Antarctique Belge. Bulletin Academie Royale Belgique 11: 814–820.

Koehler R (1901) Expédition Antarctique Belge. Résultats du voyage de SY Belgica en 1897–1898–1899, sous le commandement de A. de Gerlache de Gomery. Zoologie. Echinides et Ophiures. Buschmann, Anvers, 42 pp.

Koehler R (1907) Astéries, Ophiures et Echinides recueillis dans les mers australes par la» Scotia»(1902–1904). Zoologischer Anzeiger 32: 140–147.

Koehler R (1911) Astéries, Ophiures, et Échinides de l’Expédition Antarctique Anglaise de 1907–1909. British Antarctic Expedition 1907–1909, Reports on the Scientific Investigations 2 (Biology) 4: 25–66.

Koehler R (1912) Echinodermes (Asteries, Ophiures et Echinides). Deuxième Expédition Antarctique Française 1908–1910, 277 pp.

Koehler R (1912) Échinodermes nouveaux recueillis dans les mers antarctiques par le “Pourquoi Pas?” (Astéries, Ophiures et Échinides). Zoologischer Anzeiger 39: 151–163.

Koehler R (1926) Echinodermata Echinoidea. In: Harrison L (Ed.) Scientific Reports, series C, Zoology and Botany, Australasian Antarctic Expedition, 1911–1914, Under the Leadership of Sir Douglas Mawson, D. Sc., B.E., F.R.S Alfred J. Kent, Gove., 1–134.

Lamarck JBM de (1816) Histoire Naturelle des Animaux sans Vertèbres, présentant les caractères géneraux et particuliers de ces animaux, leur distribution, leur classes, leurs familles, leurs generes, et le citation des principales espèces qui s’y rapportent; précédée d’une Introduction offrant la Détermination des caractères essentiells de l’animal, sa distinction du végétal et des autres corps naturels, enfin, l’Exposition des Principes fondamentaux de la Zoologie. Tome Troisième. Verdière, 586 pp.

Lambert J (1905) Notes sur quelques Échinides éocéniques de l’Aude et de l’Hérault. In: Doncieux L (Ed.) Catalogue descriptif des fossiles nummulitiques de l’Aude et de l’Hérault. Annales de l’Université de Lyon, Nouvelle Série, I. Sciences, Médecine, 129–164.

Larrain AP (1985a) A new species of subantarctic echinoid (Echinoidea: Schizasteridae). Boletín de la Sociedad de Biología de Concepción 56: 115–119.

Larrain AP (1985b) Brachysternaster, new genus, and *Brachysternaster chesheri*, new species of Antarctic echinoid (Spatangoida, Schizasteridae). Polar Biol 4: 121–124. https://doi.org/10.1007/BF00263874

Leske NG (1778) Jacobi Theodori Klein naturalis dispositio echinodermatum, edita et descriptionibus novisque inventis et synonomis auctorem aucta. G.E.Beer, Leipzig, 278 pp.

Ling SD (2008) Range expansion of a habitat-modifying species leads to loss of taxonomic diversity: a new and impoverished reef state. Oecologia 156: 883–894. https://doi.org/10.1007/s00442-008-1043-9

Linnaeus C (1758) Systema Naturae per regna tria naturae, secundum classes, ordines, genera, species, cum characteribus, differentiis, synonymis, locis.

Lockhart SJ (2006) Molecular evolution, phylogenetics, and parasitism in Antarctic cidaroid echinoids. University of California, Santa Cruz.

Lovén SL (1871) 28 Om echinoideernas byggnad. Öfversigt af Kongl. Vetenskaps-Akademiens Förhandlingar, 1065–1111 pp.

Madon-Senez C (2002) A new species of marsupiate Antarctic echinoid: *Amphipneustes davidi* (Echinodermata: Spatangoida: Schizasteridae). Proceedings of the Biological Society of Washington 115: 51–56.

Markov AV (1991) The most deep-sea representative of the family Schizasteridae (Echinoidea. Zoologicheskii Zhurnal 70: 153–155.

McKnight DG (1968) Additions to the echinoid fauna of New Zealand. New Zealand. Journal of marine and freshwater Research 2: 90–110. https://doi.org/10.1080/00288330.1968.9515229

McKnight DG (1969) An Outline Distribution of the New Zealand Shelf Fauna. Benthos Survey, Station List, and Distribution of the Echinoidea. New Zealand Oceanographic Institute Memoir 47: 9–89.

McKnight DG (1974) Some echinoids new to New Zealand waters. New Zealand Oceanographic Institution Records 2: 25–44.

Meijere JCH de (1904) Die Echinoidea der Siboga-Expedition. Siboga Expeditie 43: 1–252.

Meissner M (1900) Echinoideen. L. Friedrichsen & Company.

Michelin H (1862) Annex A. Echinides et Stellerides. In: Maillard L (Ed.) Notes sur l’Île de la Réunion (Bourbon). Palais Royal, Galerie D’Orléans, Paris.

Milne Edwards G (1836) Les Zoophytes. In: Cuvier G (Ed.) Le règne animal distribué d’après son organisation, pour servir de base à l’histoire naturelle des animaux et d’introduction à l’anatomie comparée.

Mironov AN (1974) Pourtalesiid sea urchins of the Antarctic and Subantarctic (Echinoidea: Pourtalesiidae). Trudy Instituta Okeanologii Akademii Nauk SSSR 98: 240–252.

Mironov AN (1978) [Meridosternous echinoids (Echinoidea: Meridosternina) collected during the 16th cruise of the R/V ’Dm. Mendeleev’]. Trudy Instituta Okeanologii, Akademii Nauk SSSR 113: 208–226.

Mironov AN (2008) Pourtalesiid sea urchins (Echinodermata: Echinoidea) of the northern Mid-Atlantic Ridge. Marine Biology Research 4: 3–24. https://doi.org/10.1080/17451000701847863

Molina GI (1782) Saggito sulla storia naturale del Chili. 4, Animali del Chili. 367 pp. https://doi.org/10.5962/bhl.title.62689

Mooi R, David B (1996) Phylogenetic analysis of extreme morphologies: deep-sea holasteroid echinoids. Journal of Natural History 30: 913–953. https://doi.org/10.1080/00222939600770501

Mooi R, David B, Fell FJ, Chone T (2000) Three new species of bathyal cidaroids (Echinodermata: Echinoidea) from the Antarctic region. Proceedings of the Biological Society Washington 113: 224–237.

Mortensen T (1904) On some Echinothurids from Japan and the Indian Ocean. Journal of Natural History 14: 81–93. https://doi.org/10.1080/03745480409442975

Mortensen T (1904) The Danish Expedition to Siam 1899–1900. III. Echinoidea (1). Kongelige Danske Videnskabelige Selskabs. Skrifter, Serie 7: 1–124.

Mortensen T (1905) Some new species of Echinoidea. Videnskabelige Meddelelser fra den naturhistoriske Forening i Kjøbenhavn 6: 241–243.

Mortensen T (1909) Die Echinoiden der Deutschen Südpolar-Expedition 1901-1903. In: Drygalski EV (Ed.) Deutsche Südpolar-Expedition 1901-1903 im Auftrage des Reichsamtes des Innern, XI. Band, Zoologie III. Band, Heft I. Georg Reimer, Berlin, 1–114.

Mortensen T (1910) The Echinoidea of the Swedish South polar expedition. In: Nordenskjöld O (Eds) Wissenschaftliche Ergebnisse der Schwedischen Südpolar Expedition. Lithographisches Institut, Stockholm, 1–105. https://doi.org/10.5962/bhl.title.82333

Mortensen T (1921) Echinoderms of New Zealand and the Auckland-Campbell Islands 1. Echinoidea. In: Mortensen T (Ed.) Papers from Dr Th. Mortensen’s Pacific Expedition 1914–16. Videnskabelige Meddelesser Dansk Naturhistorisk Forening i Kobenhaven, 139–197.

Mortensen T (1925) Echinoderms of New Zealand and the Auckland-Campbell Islands. III-V. Asteroidea, Holothuroidea and Crinoidea. In: Mortensen T (Ed.) Papers from Dr. Th. Mortensen’s Pacific Expedition 1914 1916. Videnskabelige Meddelelser fra Dansk naturhistorisk Forening i København, 261–420.

Mortensen T (1928) A Monograph of the Echinoidea. I. Cidaroidea. C. A. Reitzel & Oxford University Press, Copenhagen & London, 551 pp.

Mortensen T (1930) Some new Japanese echinoids. Annotationes Zoologicae Japonenses 12: 387–402.

Mortensen T (1936) Echinoidea and Ophiuroidea. Discovery Reports 12: 199–348. https://doi.org/10.5962/bhl.part.8051

Mortensen T (1939) New Echinoida (Aulodonta). Preliminary Notice. Videnskabelige Meddelelsar Dansk Naturhistoriske Forening i København 103: 547–550.

Mortensen T (1943) A monograph of the Echinoidea Part III.2, Camarodonta 1 Orthopsidæ, Glyphocyphidæ, Temnopleuridæ and Toxopneustidæ. C. A. Reitzel, Copenhagen, 553 pp.

Mortensen T (1950a) British Australian New Zealand Antarctic Research Expedition, 1929–1931, Echinoidea. BANZAR Expedition Reports, Series B (Zoology and Botany) 4: 287–310.

Mortensen T (1950b) New Echinoidea (Spatangoida). Preliminary notice. Videnskabelige Meddelelsar Dansk Naturhistoriske Forening i København 112: 157–163.

Oyarzún ST, Marín SL, Valladares C, Iriarte JL (1999) Reproductive cycle of *Loxechinus albus* (Echinodermata: Echinoidea) in two areas of the Magellan Region (53°S, 70–72°W), Chile. Scientia Marina 63: 439–449. https://doi.org/10.3989/scimar.1999.63s1439

Pawson DL (1964) The echinoid genus Caenopedina in New Zealand. Transactions of the Royal Society of New Zealand, Zoology 5: 63–66.

Pawson DL (1968) Echinoderms. Australian Natural History 16: 129–133.

Pennant T (1777) British Zoology. London 1777 36: 1–154.

Philippi RA (1845) Beschreibung einiger neuer Echinodermen nebst kritischen Bemerckungen über einige weniger bekannte Arten. Archiv für Naturgeschichte 11: 344–359.

Philippi RA (1857) Vier neue Echinodermen des Chilenischen Meeres. Archiv für Naturgeschichte 23: 130–34.

Pomel A (1883) Classification méthodique et Genera des Echinides vivants et fossiles. Adolphe Jourdan, Alger, 131pp. https://doi.org/10.5962/bhl.title.11272

Poulin É, Féral J-P (1995) Pattern of spatial distribution of a brood-protecting schizasterid echinoid, *Abatus cordatus*, endemic to the Kerguelen Islands. Marine Ecology Progress Series: 179–186. https://doi.org/10.3354/meps118179

Ríos C, Mutschke E (1999) Community structure of intertidal boulder-cobble fields in the Straits of Magellan, Chile. Scientia Marina 63: 193–201. https://doi.org/10.3989/scimar.1999.63s1193

Rowe FWE (1989) Nine New Deep-Water Species of Echinodermata from Norfolk Island and Wanganella Bank, northeastern Tasman Sea, with a Checklist of the Echinoderm Fauna. Proceedings of The Linnean Society of New South Wales 111: 257–291.

Rowe FWE, Hoggett AK (1986) The cidarid echinoids (Echinodermata) of New South Wales. Proceedings of the Linnean Society of NSW 108: 225–261.

Stefanini G (1912) Osservazioni sulla distribuzione geografica sulle origini e sulla filogenesi degli Scutellidae. Bolletino della Società Geologica Italiana 30: 739–754.

Studer T (1876) Echinodermen aus dem antarktischen Meere und zwei neue Seeigel von den Papua-Inseln, gesammelt auf der Reise S.M.S. Gazelle um die Erde. Monatsbericht der königlich preussischen Akademie der Wissenschaften zu Berlin: 452–465.

Tenison-Woods JE (1879) On some new Australian Echini. Proceedings of the Linnean Society of New South Wales: 282–291.

Thomson CW (1871) On the Echinidea of the “Porcupine” Deep-Sea Dredging-Expeditions. Proceedings of the Royal Society of London 20: 491–497. https://doi.org/10.1098/rspl.1871.0095

Thomson CW (1872) Notice of a New Family of the Echinodermata. Proceedings of the Royal Society of Edinburgh 7: 615–617. https://doi.org/10.1017/S0370164600042759

Thomson CW (1876) Notice of some Peculiarities in the Mode of Propagation of certain Echinoderms of the Southern Sea. Zoological Journal of the Linnean Society 13: 55–79. https://doi.org/10.1111/j.1096-3642.1876.tb00209.x

Thomson CW (1878) 1 The Voyage of the» Challenger»: The Atlantic; a Preliminary Account of the General Results of the Exploring Voyage of HMS» Challenger» During the Year 1873 and the Early Part of the Year 1876. Macmillan and Company, 424 pp.

Troschel FH (1851) Ueber die Gattung Tripylus. Archiv für Naturgeschichte 17: 67–74.

Troschel FH (1872) Die Familie der Echinocidariden (1). Archiv für Naturgeschichte 38: 293–356.

Tyberghein L, Verbruggen H, Pauly K, Troupin C, Mineur F, De Clerck O. (2012) Bio-ORACLE: a global environmental dataset for marine species distribution modelling. Global Ecology and Biogeography 21(2): 272–281. https://doi.org/10.1111/j.1466-8238.2011.00656.x

Valenciennes A (1846) Zoophytes. In: du Petit-Thouars A (Ed.)Voyage autour de monde sur la frégate La Vénus, pendant les années 1836–1839. Atlas de Zoologie. Gide et Cie, Paris.

Verrill AE (1876) Contribution to the natural history of Kerguelen Island. Annelids and Echinoderms. Bulletin United States National Museum 3: 64–75.

Yoshiwara S (1898) Preliminary notice of new Japanese Echinoids. Annotationes Zoologicae Japonenses 2: 57–61.
